# Data on synthesis and characterization of new p-nitro stilbene Schiff bases derivatives as an electrochemical DNA potential spacer

**DOI:** 10.1016/j.dib.2020.105568

**Published:** 2020-04-22

**Authors:** Nur Zafirah Mohd Izham, Hanis Mohd Yusoff, Irshad Ul Haq Bhat, Tomoaki Endo, Hiroshi Fukumura, Eunsang Kwon, Shin-Ichiro Yoshida, Asnuzilawati Asari, Uwaisulqarni M. Osman, Mohd Sukeri Mohd Yusof

**Affiliations:** a Faculty of Science and Marine Environment, Universiti Malaysia Terengganu, 21030 Kuala Nerus, Terengganu, Malaysia; b Advanced Nano Materials (ANoMa) Research Group, Faculty of Science and Marine Environment, Universiti Malaysia Terengganu, 21030 Kuala Nerus, Terengganu, Malaysia; c Sendai National College of Technology, 48 Nodayama, Madeshima-Shiote, Natori-shi, Miyagi 981-1239, Japan; d Graduate School of Science, Tohoku University, Aramaki Aoba, Aoba-ku, Sendai 980-8578, Miyagi, Japan; e Research and Analytical Center for Giant Molecules, Graduate School of Science, Tohoku University, Aramaki Aoba, Aoba-ku, Sendai 980-8578, Miyagi, Japan

**Keywords:** Aldehyde, Imine, Heck reaction, Schiff base derivatives, Stilbene, Spacer

## Abstract

The structural investigation of synthesized compounds can be carried out by various spectroscopic techniques. It is an important prospect in order to elucidate the structure of the desired products before being further utilized. The preparation of new p-nitro stilbene Schiff base derivatives as an electrochemical DNA potential spacer was synthesized using (E)-4-(4-nitrostyryl)aniline from Heck reaction with aldehydes in ethanolic solution. The data presented here in this article contains FTIR, UV-Vis and ^1^H and ^13^C NMR of (E)-4-(4-nitrostyryl)aniline and nitrostyryl aniline derivatives.

Specifications table

**Table:** 

Subject	Chemistry
Specific subject area	Spectroscopy
Type of data	Table Figure Scheme
How data were acquired	FTIR (Shimadzu IRTracer-100 Fourier Transform Infrared Spectrometer) ranges from 400 to 4000 cm^−1^ by using single reflection ATR; UV-Vis (double beam Shimadzu UV-1800 spectrophotometer) ranges from 190 to 800nm by using acetonitrile as solvent in 1cm^3^ cuvette; ^1^H and ^13^C nuclear magnetic resonance (NMR) spectra (Bruker Avance II 400 spectrophotometer) using deuterated dimethylsulfoxide (DMSO-d_6_) as solvent.
Data format	Raw and analysed
Parameters for data collection	All compounds were synthesized at room temperature.
Description of data collection	The synthesized compounds from Heck and Schiff base reactions were characterized by spectroscopic method for establishing the structure of compounds.
Data source location	Faculty of Science and Marine Environment, Universiti Malaysia Terengganu, 21030 Kuala Nerus, Terengganu, Malaysia.
Data accessibility	http://dx.doi.org/10.17632/fw86ngty6c.1

## Value of the data

• The following data shows new derivatives synthesized from (E)-4-(4-nitrostyryl)aniline, which are important in structure elucidation and are useful to researchers who are developing spacer to be applied on substrate used in electrochemical DNA sensor.
• For future investigations, the details in these data could be used for comparison study with previous spacers, which in turn could help in further understanding of the significant role of substituent attached at the (nitrostyryl)aniline terminal end.
• The details in this data could be extended towards other field such as for antibacterial study and other biological application as many studies has reported that imine structure could inhibit the bacterial growth.

## 1 Data

**Scheme 1:**
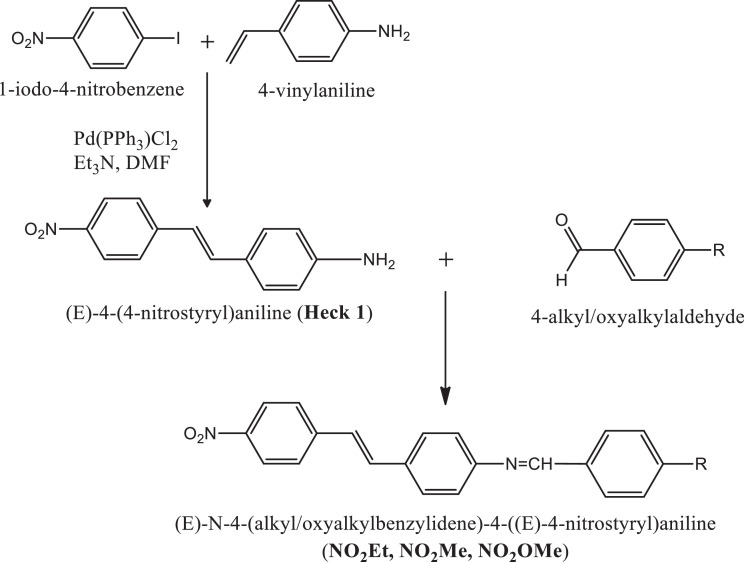
Overall reaction scheme.

**Fig. 1:**
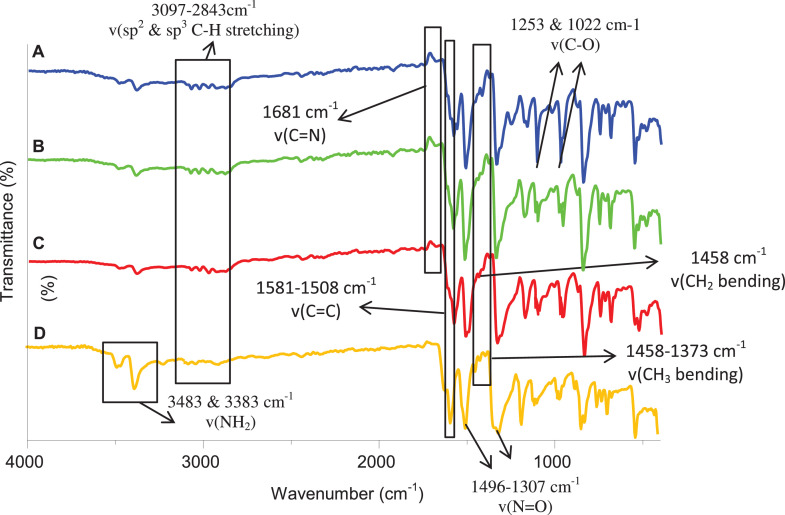
Infrared spectra of compounds formed. (A) Heck 1, (B) NO_2_Et, (C) NO_2_Me and (D) NO_2_OMe.

**Fig. 2:**
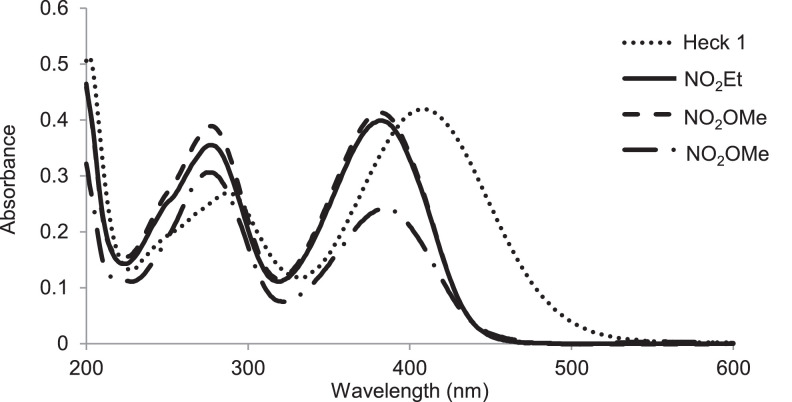
UV-Visible spectra of Heck 1, NO_2_Et, NO_2_Me and NO_2_OMe in acetonitrile, respectively.

**Fig. 3:**
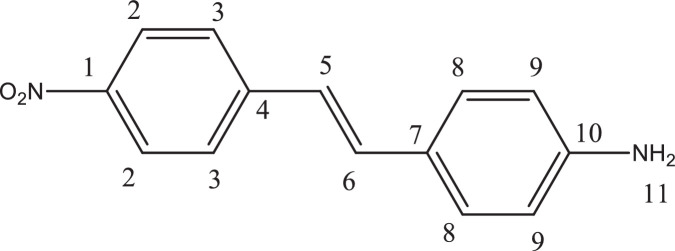
Heck 1.

**Fig. 4:**
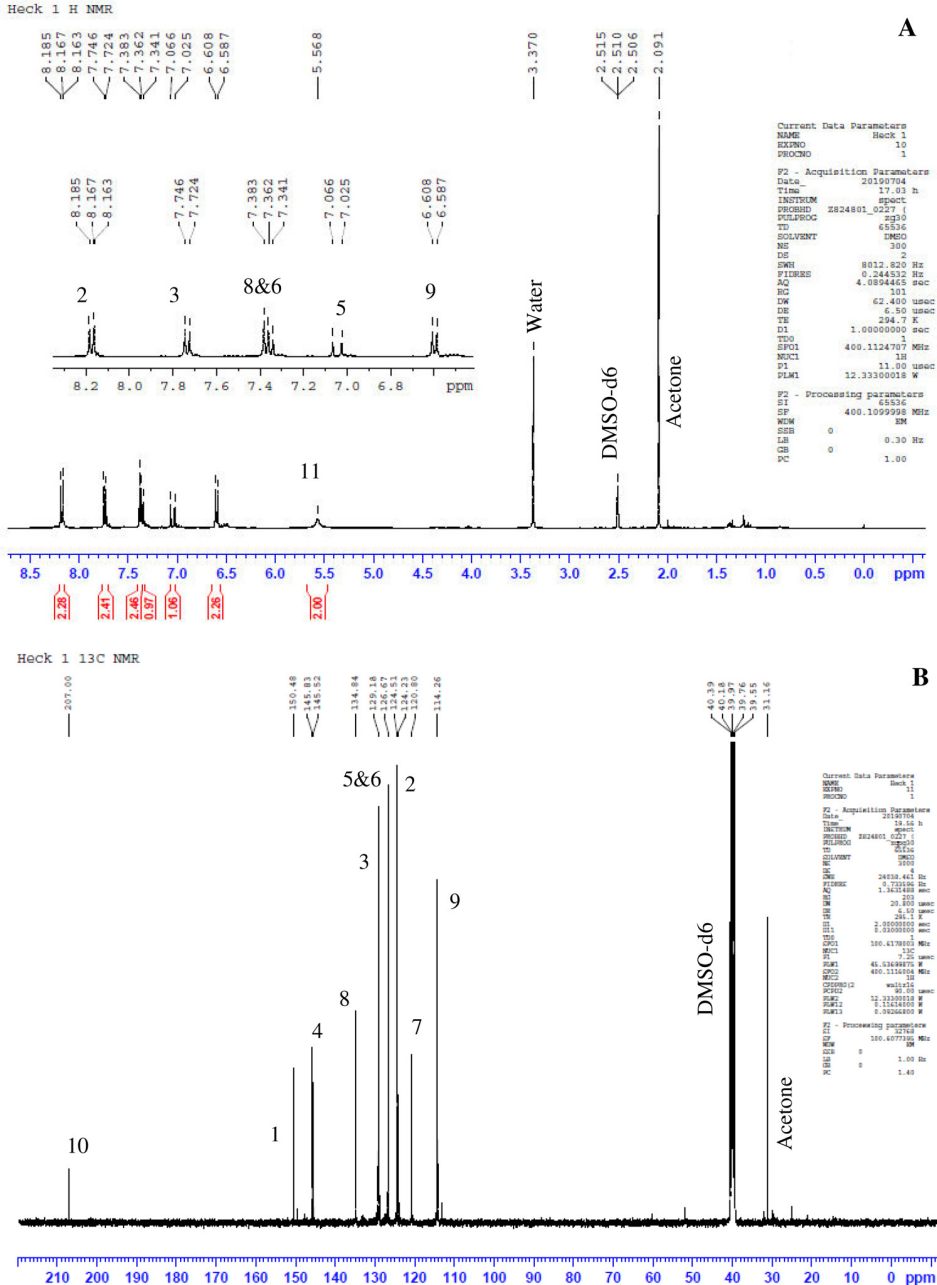
(A) ^1^H and (B) ^13^C NMR of Heck 1.

**Fig. 5:**
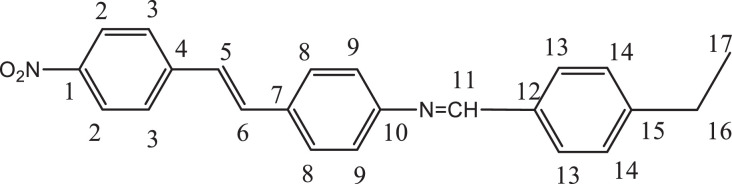
NO_2_Et.

**Fig. 6:**
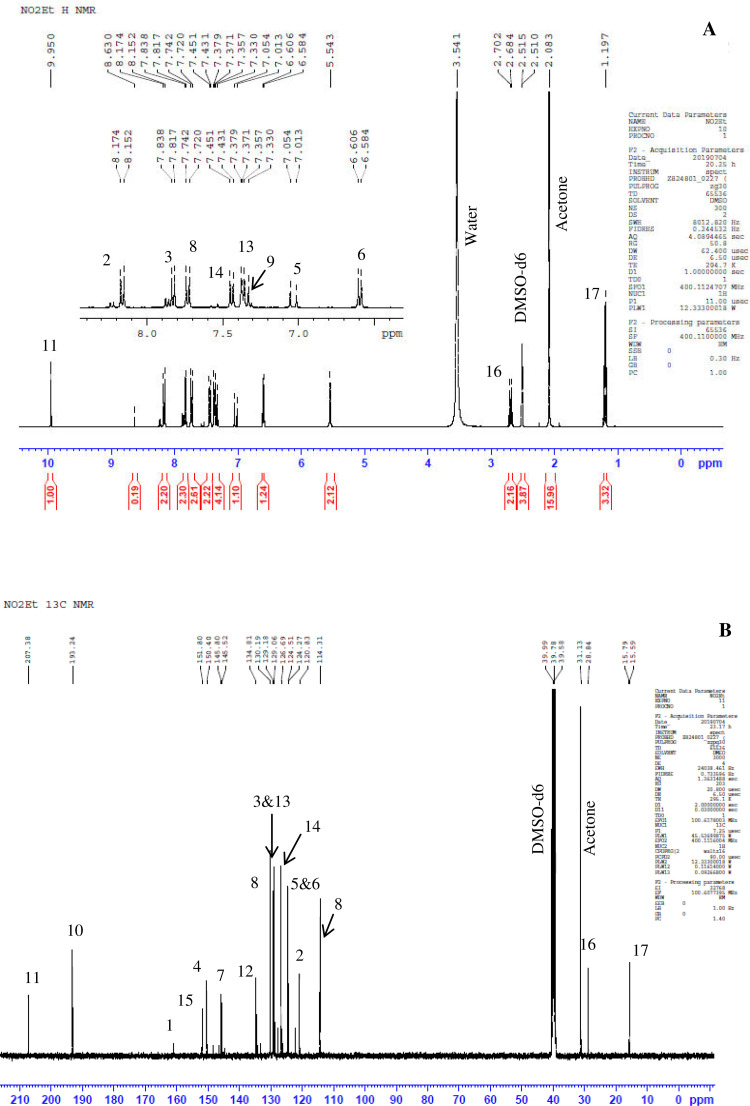
(A) ^1^H and (B) ^13^C NMR of NO_2_Et.

**Fig. 7:**
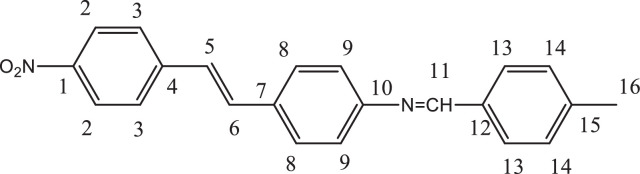
NO_2_Me.

**Fig. 8:**
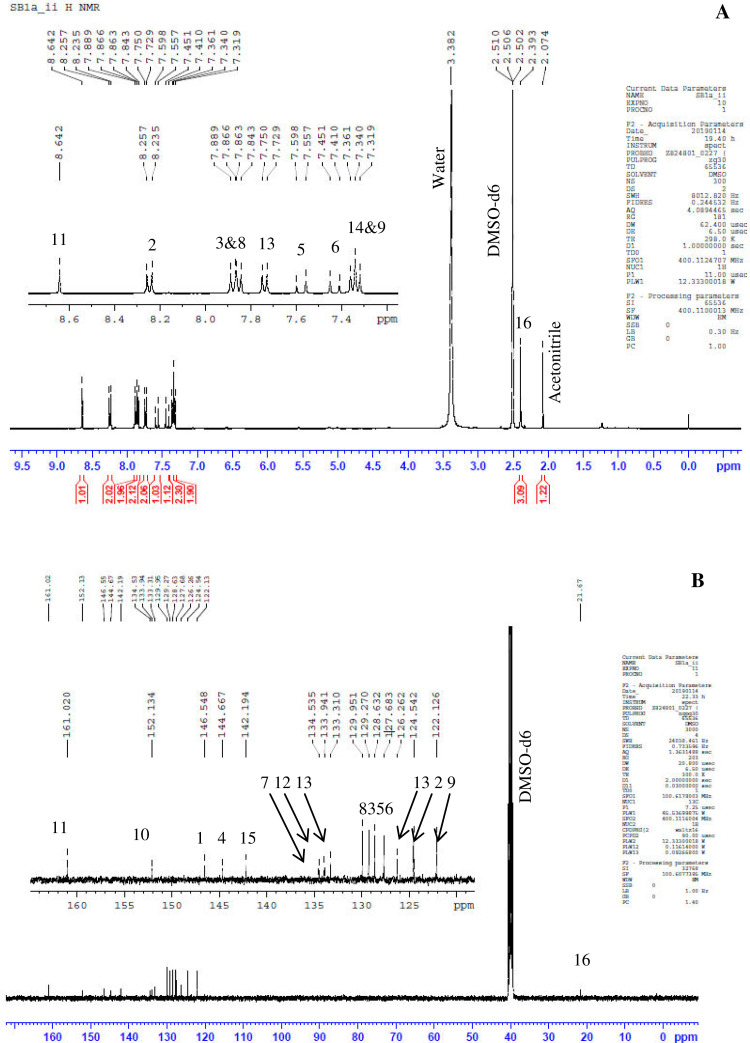
(A) ^1^H and (B) ^13^C NMR of NO_2_Me.

**Fig. 9:**
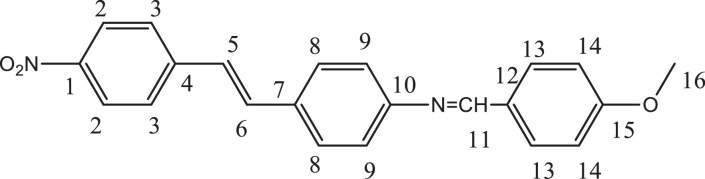
NO_2_Ome.

**Fig. 10:**
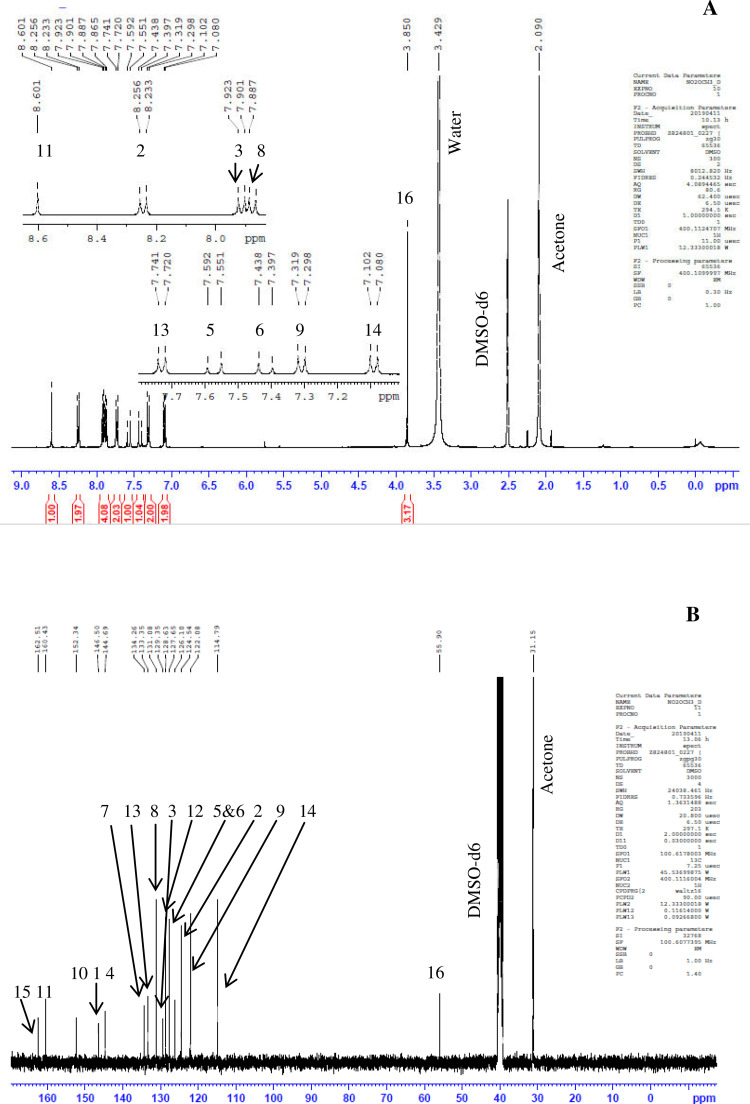
(A) ^1^H and (B) ^13^C NMR of NO_2_OMe.

Previously, Hassan et al. (2018) had synthesized several stilbene Schiff base derivatives with various alkyl chain lengths [1], [2], [3]. This paper deals about the identification and characterization of Heck product and three (nitrostyryl)aniline derivatives of the same length but with different terminal end as a comparison with alkyl terminal end. This paper deals about the identification and characterization of Heck product and three (nitrostyryl)aniline derivatives. It describes the preparation of samples prior the spectroscopy measurements which presented accordingly. Scheme 1 describes the overall reaction involved in producing (E)-N-(4-ethylbenzylidene)-4-((E)-4-nitrostyryl)aniline (NO_2_Et), (E)-N-(4-methylbenzylidene)-4-((E)-4-nitrostyryl)aniline (NO_2_Me) and (E)-N-(4-methoxybenzylidene)-4-((E)-4-nitrostyryl)aniline (NO_2_OMe), which includes Heck and Schiff base reactions. Table 1 and Fig. 1 describe the comparative assessment of FTIR of all the samples. Whereas, Table 2 and Fig. 2 describe the UV-vis data comparison of four products produced. Tables 3 and 4 tabulate the ^1^H and ^13^C NMR of (E)-4-(4-nitrostyryl)aniline (Heck 1) and three imine products. Figs. 4, 6, 8 and 10 display the images of NMR spectra of Figs. 3, 5, 7 and 9 that are being studied, respectively.

**Table 1:** Frequencies of selected bands of diagnostic importance from the IR spectra of Heck 1 and nitrostyryl aniline Schiff bases derivatives.

Compound frequency (cm^−1^)				Assignment
Heck 1	NO_2_Et	NO_2_Me	NO_2_OMe	
3483 & 3383	-	-	-	NH_2_
1496 & 1307	1458 & 1334	1419 & 1338	1458 & 1338	N=O
3070-2900	3074-2877	3097-2868	3074-2843	C-H stretching
-	1458	-	-	CH_2_ bending
-	1373	1377	1373	CH_3_ bending
-	1681	1681	1681	C=N
-	-	-	1253 & 1022	C-O stretching
1581	1516	1516	1508	C=C alkene

**Table 2:** Wavelengths of UV-visible spectra values of Heck 1 and nitrostyryl aniline Schiff bases derivatives.

Peak	Compound wavelength, nm (absorbance)				Assignment
	Heck 1	NO_2_Et	NO_2_Me	NO_2_OMe	
1	408.0 (0.42)	381.50 (0.399)	382.5 (0.413)	384.5 (0.242)	R-NO_2_ and C=N (n → π*)
2	288.0 (0.27)	276.50 (0.355)	277.0 (0.389)	276.5 (0.306)	C=C aromatic (*π* → π*)

**Table 3:** Chemical shifts of ^1^H NMR values of Heck 1 and nitrostyryl aniline Schiff bases derivatives.

Signal	^1^H NMR chemical shifts of compounds (ppm)				Assignment
	Heck 1	NO_2_Et	NO_2_Me	NO_2_OMe	
2	8.17, 8.19 (d)	8.15, 8.17 (d)	8.24, 8.26 (d)	8.23, 8.26 (d)	Aromatic
3	7.72, 7.75 (d)	7.82, 7.84 (d)	7.87, 7.89 (d)	7.90, 7.92 (d)	Aromatic
5	7.03, 7.07 (d)	7.01, 7.05 (d)	7.56, 7.60 (d)	7.55, 7.59 (d)	C=C
6	7.34 (s)	6.58, 6.61 (d)	7.41, 7.45 (d)	7.40, 7.44 (d)	C=C
8	7.36, 7.38 (d)	7.72, 7.74 (d)	7.84, 7.86 (d)	7.89, 7.87 (d)	Aromatic
9	6.59, 6.61 (d)	7.33 (s)	7.32 (s)	7.30, 7.32 (d)	Aromatic
11	5.57 (s)	9.95 (s)	8.64 (s)	8.60 (s)	NH2
13	-	7.36, 7.38 (d)	7.41, 7.45 (d)	7.72, 7.74 (d)	Aromatic
14	-	7.43, 7.45 (d)	7.34, 7.36 (d)	7.08, 7.10 (d)	Aromatic
16	-	2.68, 2.70 (d)	2.39 (s)	3.85 (s)	Alkane
17	-	1.20 (s)	-	-	Alkane

**Table 4:** Chemical shifts of ^13^C NMR values of Heck 1 and nitrostyryl aniline Schiff bases derivatives.

Signal	^13^C NMR chemical shifts of compounds (ppm)				Assignment
	Heck 1	NO_2_Et	NO_2_Me	NO_2_OMe	
1	150.48	161.01	146.55	146.50	Aromatic
2	124.51	120.83	124.54	124.54	Aromatic
3	129.18	129.18	129.27	129.35	Aromatic
4	145.83	150.40	144.67	144.69	Aromatic
5 & 6	126.67	124.51	127.68	127.65	C=C
7	120.80	145.80	134.54	134.26	Aromatic
8	134.84	130.19	129.95	131.08	Aromatic
9	114.26	114.31	122.13	122.08	Aromatic
10	207.00	193.24	152.13	152.34	Aromatic
11	-	207.38	161.02	160.43	Imine
12	-	134.81	133.94	128.63	Aromatic
13	-	129.06	133.31	133.35	Aromatic
14	-	126.69	126.26	114.79	Aromatic
15	-	151.80	142.19	162.51	Aromatic
16	-	28.84	21.67	55.90	Alkane
17	-	15.79	-	-	Alkane

## 2 Experimental Design, Materials, and Methods

### 2.1 Materials

4-vinylaniline, 4-ethylbenzaldehyde, p-tolualdehyde and p-anisaldehyde (98%) were purchased from Acros Organics; N,N-dimethylformamide and dichloromethane were from Fisher Scientific, while 1-iodo-4-nitrobenze (98%), trimethylamine and ethanol absolute were purchased from Sigma-Aldrich, R&M Chemicals and HmbG Chemicals, respectively. All chemicals were used as received.

### 2.2 Instrumentation

The structure of synthesized products was established by spectral data obtained by different spectroscopic instruments. FTIR, UV-Vis and NMR were recorded by Shimadzu IRTracer-100 Fourier Transform Infrared Spectrometer, double beam Shimadzu UV-1800 spectrophotometer and Bruker Avance II 400 spectrophotometer, respectively. Reaction progresses of the compounds were monitored by Thin layer chromatography (TLC) using silica gel 60 F254, 0.25 mm thick plastic plates [1,3].

### 2.3 Synthesis of (E)-4-(4-nitrostyryl)aniline

A substituted aryl halide (1 mol) was added to a mixture of N, N-dimethylformamide (3 ml) and 4-vinylaniline (1 mol, 0.4 g), trimethylamine (3 ml) as base and bis(triphenylphosphine)palladium chloride (40 mg) as catalyst in a three-neck round bottom flask. The resulting mixture was stirred well and refluxed for 24 hours at 70-80°C. The completion of reaction was monitored by TLC. The reaction mixture was filtered and thoruoghly washed by dichloromethane. Next, the solvent was evaporated by the rotary evaporator and run for column chromatography in order to obtain the the product of (E)-4-(4-nitrostyryl)aniline (Heck 1) (Fig. 3) as previously produced by [3].

### 2.4 Synthesis of Schiff bases derivatives

Schiff base method of [4] was modified to produce imine compounds. In a Dean-stark flask 1 mol of the synthesized product from Heck reaction dissolved in ethanol (50 ml), 0.1 ml of commercial aldehyde (p-tolualdehyde or p-ansaldehyde) in ethanol (10 ml) was added at 60°C. The reaction mixture was refluxed for 2 hours at 80°C. The reaction mixture was cooled down and the product was precipitated. The yielded product was filtered off and recrystallized from hot acetonitrile [5]. Nitrostyryl aniline derivatives (Figs. 2, 7, 9) produced were weighed and the percentage yield was recorded.

### 2.5 Complete characterization description of the four compounds

*(E)-4-(4-nitrostyryl)aniline (Heck 1):* Brick-red powder; ATR: 3483 & 3388 (NH_2_, stretching), 1496 & 1307 (N=O), 3070-2900 (C-H stretching,)1581 (C=C alkene); UV–Vis spectrum [ACN, λmax nm (log ɛ, L mol^−1^ cm^−1^)]: 408.00 (4.62), 288.00 (4.43); ^1^H NMR (400MHz, DMSO-d_6_): δ 8.19-6.59 (d, 10H), 5.57 (s, 2H); ^13^C NMR (100MHz, DMSO-d_6_): δ 207.0, 150.48, 145.83, 129.18, 126.67, 124.51, 120.80, 114.26.

*(E)-N-(4-ethylbenzylidene)-4-((E)-4-nitrostyryl)aniline* (*NO_2_Et*): Yellow flakes; ATR: 1458 & 1334 (N=O), 3074-2877 (C-H, stretching), 1458 (CH_2_, bending), 1373 (CH_3_, bending), 1681 (C=N), 1516 (C=C alkene): UV–Vis spectrum [ACN, λmax nm (log ɛ, L mol^−1^ cm^−1^)]: 381.50 (4.60), 276.50 (5.55); ^1^H NMR (400MHz, DMSO-d_6_): δ 9.95 (s, 1H), 8.17-6.58 (d, 14H), 2.70-2.68 (d, 2H), 1.20 (s, 3H);); ^13^C NMR (100MHz, DMSO-d_6_): δ 207.38, 193.24, 161.01, 151.80, 150.40, 145.80, 134.81, 130.19, 129.18, 126.69, 129.06, 124.51, 120.83, 114.31, 28.84, 15.79.

*(E)-N-(4-methylbenzylidene)-4-((E)-4-nitrostyryl)aniline* (*NO_2_Me*): Yellow flakes; ATR: 1419 & 1338 (N=O), 3097-2868 (C-H, stretching), 1377 (CH_3_, bending), 1681 (C=N), 1516 (C=C alkene); UV–Vis spectrum [ACN, λmax nm (log ɛ, L mol^−1^ cm^−1^)]: 382.50(4.62), 277.00 (4.59); ^1^H NMR (400MHz, DMSO-d_6_): δ 8.64 (s, 1H), 8.26-7.32 (d, 14H), 2.39 (s, 3H); ^13^C NMR (100MHz, DMSO-d_6_): δ 161.02, 152.13, 146.55, 144.67, 142.19, 134.54, 133.94, 133.31, 129.95, 129.97, 127.68, 126.26, 124.54, 122.13, 21.67.

*(E)-N-(4-methoxybenzylidene)-4-((E)-4-nitrostyryl)aniline* (*NO_2_OMe*): Yellow flakes; ATR: 1458 & 1338 (N=O), 3074-2843 (C-H, stretching), 1373 (CH3, bending), 1681 (C=N), 1253 & 1022 (C-O, stretching), 1508 (C=C alkene); UV–Vis spectrum [ACN, λmax nm (log ɛ, L mol^−1^ cm^−1^)]: 384.5 (4.38), 276.5 (4.49); ^1^H NMR (400MHz, DMSO-d_6_): δ 8.60 (s, 1H), 8.26-7.32 (d, 14H), 2.39 (s, 3H); ^13^C NMR (100MHz, DMSO-d_6_): δ 162.51, 160.42, 152.34, 146.50, 144.69, 134.26, 133.35, 131.08, 129.35, 128.63, 127.65, 124.54, 122.08, 14.79, 55.90.

## Appendix Supplementary materials

Image, application 1
